# Metabolic and lifestyle factors in relation to senile cataract: a Mendelian randomization study

**DOI:** 10.1038/s41598-021-04515-x

**Published:** 2022-01-10

**Authors:** Shuai Yuan, Alicja Wolk, Susanna C. Larsson

**Affiliations:** 1 Unit of Cardiovascular and Nutritional Epidemiology, Institute of Environmental Medicine, Karolinska Institutet, Nobels väg 13, 17177 Stockholm, Sweden; 2 Unit of Medical Epidemiology, Department of Surgical Sciences, Uppsala University, Uppsala, Sweden

**Keywords:** Eye diseases, Genetics, Risk factors

## Abstract

We conducted a Mendelian randomization study to determine the associations of body mass index (BMI), type 2 diabetes (T2D), systolic blood pressure (SBP), coffee and alcohol consumption and smoking initiation with senile cataract. Independent single nucleotide polymorphisms associated with the metabolic and lifestyle factors at the *p* < 5 × 10^–8^ were selected as instrument variables. Summary-level data for senile cataract were obtained from the FinnGen consortium (20,157 cases and 154,905 non-cases) and UK Biobank study (6332 cases and 354,862 non-cases). Higher genetically predicted BMI and SBP and genetic predisposition to T2D and smoking initiation were associated with an increased risk of senile cataract. The combined odds ratios were 1.19 (95% confidence interval (CI) 1.09–1.29; *p* < 0.001) per one standard deviation increase in BMI (~ 4.8 kg/m^2^), 1.13 (95% CI 1.04–1.23; *p* = 0.004) per 10 mmHg increase in SBP, 1.06 (95% CI 1.03–1.09; *p* < 0.001) per one unit increase in log-transformed odds ratio of T2D, and 1.19 (95% CI 1.10–1.29; *p* < 0.001) per one standard deviation increase in prevalence of smoking initiation. Genetically predicted coffee consumption showed a suggestive association with senile cataract (odds ratio per 50% increase, 1.18, 95% CI 1.00–1.40; *p* = 0.050). This study suggests causal roles of obesity, T2D, SBP and smoking in senile cataract.

## Introduction

Senile cataract, known as age-related cataract, affects ~ 17% of the population^1^ and causes over 50% of vision loss^2^ and large disease and economic burdens^3,4^ globally. Metabolic syndrome components^5–7^ and certain lifestyles, such as coffee^8,9^ and alcohol consumption^10–12^ and cigarette smoking^13^, have been associated with risk of senile cataract in observational studies. Nevertheless, data on the associations of coffee^8,9,14^ and alcohol^10–12,14^ consumption with senile cataract are conflicting and the causality of these observed associations remains undetermined due to possible limitations, such as residual confounding and other biases, embedded in observational studies. An appraisal of the causality of the associations of metabolic and lifestyle factors with senile cataract risk may provide clues for the primary prevention of senile cataract.

Mendelian randomization (MR) is an epidemiological approach that can strengthen the causal inference by leveraging genetic variants as instrumental variables for an exposure^15^. The method can minimize the residual confounding since the genetic variants are randomly assorted at conception and thus unassociated with self‐selected lifestyle and environmental factors^15^. Here, we conducted a MR study to determine the associations of three metabolic traits, including body mass index (BMI), type 2 diabetes and systolic blood pressure (SBP), and three lifestyle factors, including coffee and alcohol consumption and smoking, with risk of senile cataract.

## Materials and methods

All methods used in this study were carried out in accordance with relevant guidelines and regulations^16^. All studies included in cited genome‐wide association studies had been approved by a relevant review board. All participants had provided informed consent. The present MR analyses were approved by the Swedish Ethical Review Authority (2019‐02793).

### Genetic instrument selection

Single nucleotide polymorphisms (SNPs) associated with BMI^17^, type 2 diabetes^18^, SBP^19^, coffee^20^ and alcohol^21^ consumption, and smoking initiation^21^ were identified at the genome-wide significance level (*p* < 5 × 10^–8^) from corresponding large-scale meta-analyses of genome-wide association studies. Linkage disequilibrium across SNPs for one exposure were estimated in the European population of 1000 Genomes as reference panel. We defined independent SNPs by *r*^2^ < 0.01 and clump distance > 10,000 kb and used these SNPs as instrumental variables in MR analysis. Detailed information on used genetic studies and consortia are displayed in Table 1.

**Table 1:** Characteristics of used studies and consortia.

Exposure or outcome	Unit	SNPs ^a^	R^2^ (%)	F statistic^b^	Participants	Adjustments	PubMed ID or URL^c^
Body mass index	1 SD (~ 4.8 kg/m^2^)	312	7.7	46.7/96.5	806,834 individuals of European ancestry	Age, sex and genetic 1–5 principal components	30239722
Type 2 diabetes	1-unit in prevalence of type 2 diabetes	497	NA	–	228,499 type 2 diabetes cases and 1,178,783 non-cases of multi-ancestries	Age, sex, and the first 10 genetic principal components	32541925
Systolic blood pressure	10 mm Hg	231	4.8	38.2/78.8	Up to 1,006,863 individuals of European ancestry	Age, sex, BMI, genotyping chips	30224653
Coffee consumption	50% change (e.g., from 1 to 1.5 cups) per day	12	0.5	73.3/151.2	375,833 European-descent individuals	Age, sex, BMI, total energy, proportion of typical food intake, and 20 genetic principal components	31046077
Alcohol consumption	1 SD increase of log-transformed alcoholic drinks/week	84	0.3	6.3/12.9	941,280 European-descent individuals	Age, sex, and the first 10 genetic principal components	30643251
Smoking initiation	1 SD in prevalence of smoking initiation	314	2.4	13.7/28.3	1,232,091 European-descent individuals	Age, sex, and the first 10 genetic principal components	30643251
Senile cataract	–	–	–	–	20,157 cases and 154,905 non-cases of European ancestry	Age, sex, 10 genetic principal components, and genotyping batch	FinnGen consortium
Senile cataract	–	–	–	–	6,332 cases and 354,862 non-cases of European ancestry	Age, sex, and up to 20 genetic principal components	UK Biobank

*NA* not available, *SD* standard deviation, *SNPs* single nucleotide polymorphisms.

^a^Independent SNPs were defined by r^2^ < 0.01 and distance > 10,000 kb.

^b^F statistic in the FinnGen consortium/F statistic in the UK Biobank.

^c^FinnGen consortium (https://www.finngen.fi/fi) and UK Biobank (http://www.nealelab.is/uk-biobank).

#### Data sources for senile cataract

Summary-level data (i.e., beta coefficient and corresponding standard error) for the associations of exposure-associated SNPs with senile cataract were obtained from the UK Biobank study^22^ and the FinnGen consortium^23^. The senile cataract cases were defined by H25 in International Classification of Disease-10 (ICD-10), 3661 in ICD-9 and 37,402 in ICD-8 in FinnGen. We used data from the 4th release of the FinnGen consortium comprising 20,157 cases and 154,905 non-cases of Finnish descent after the exclusion of individuals with ambiguous gender, high genotype missingness (> 5%), excess heterozygosity (± 4 SDs), and non-Finnish ancestry. Data from the UK Biobank study were extracted from the results of the 2nd wave of genome-wide association analyses in UK Biobank, performed by the Neale lab (http://www.nealelab.is/uk-biobank). A total of 6332 cases (defined by H25 in ICD-10) and 354,862 non-cases was included in the analysis after the removal of individuals of non-European ancestry, closely related individuals (third-degree relatives or closer), individuals with sex chromosome aneuploidies, and individuals who had withdrawn consent from the UK Biobank study.

#### Statistical analysis

We aligned the SNPs based on allele letter and allele frequency. The F statistic was calculated to assess the strength of genetic instruments and the F statistic for one association > 10 was deemed good and less likely to be biased by weak instrument (Table 1). We used the multiplicative random-effects inverse-variance weighted model as the main statistical method. We examined the genetic correlation on cataract between the UK Biobank and FinnGen using the ldsc software^24,25^ and found a high consistency (*rg* = 0.92; *p* = 1.3 × 10^–9^). Thus, estimates from two data sources were combined using the fixed-effect meta-analysis method. Four sensitivity analyses, including the weighted median^26^, MR-Egger^27^, MR-PRESSO^28^, and contamination mixture^29^ methods, were performed to examine the robustness of the results and detect and correct for possible horizontal pleiotropy. The weighted median approach can provide consistent causal estimates at the presupposition that > 50% of weight comes from the valid instruments^26^. The MR-Egger regression can detect pleiotropic effects by its intercept (*p* for intercept < 0.5 indicates pleiotropy) and provide estimates after the correction for pleiotropy although it consumes statistical power^27^. The MR-PRESSO method can detect outlying SNPs and provide causal estimates after the removal of identified outliers^28^. The contamination mixture method excels at the analysis using hundreds of SNPs as instrumental variables for an exposure and allows for the presence of invalid SNPs^29^. Considering phenotypic and genetic correlations between alcohol consumption and smoking initiation^21^, we performed multivariable MR analysis^30^ to minimize mutual pleiotropy. The odds ratio (OR) of senile cataract was scaled to units listed in Table 1 for exposures. We used Cochrane’s Q value to assess the heterogeneity of estimates from SNPs in each analysis. Associations with a *p* value < 0.008 (0.05/6 outcomes) were deemed significant associations, and associations with a *p* value ≥ 0.008 and ≤ 0.05 were regarded as suggestive associations. All tests were two-sided and performed using the TwoSampleMR^31^, MR-PRESSO^28^ and MendelianRandomization^32^ packages in the R software (version 4.0.2).

## Results

**Figure 1:**
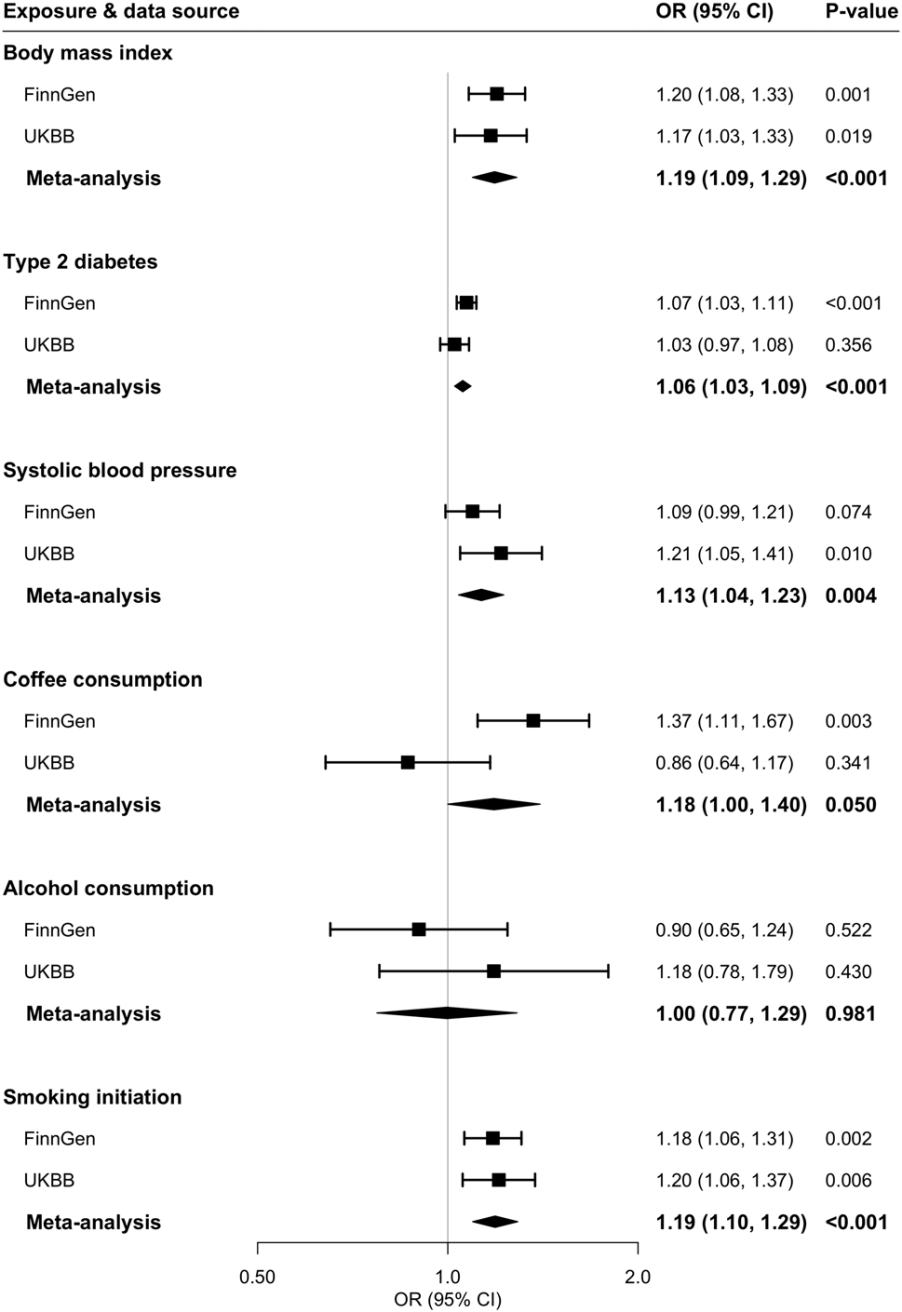
Associations of genetically predicted metabolic and lifestyle factors with senile cataract risk. *CI* confidence interval, *OR* odds ratio, *UKBB* UK Biobank. Causal estimates were obtained from random-effects inverse variance weighted method.

Among metabolic traits, higher genetically predicted BMI was associated with an increased risk of senile cataract in the FinnGen consortium, UK Biobank study and the meta-analysis (Fig. 1). A genetically predicted one standard deviation increase in BMI corresponded to a combined OR of 1.19 (95% confidence interval (CI) 1.09, 1.29; *p* < 0.001). Genetic liability to type 2 diabetes and higher genetically predicted SBP were positively associated with senile cataract in FinnGen and UK Biobank, respectively, and these associations remained directionally consistent in the other data source (Fig. 1). The combined ORs of senile cataract were 1.06 (95% CI 1.03, 1.09; *p* < 0.001) for one unit increase in genetically predicted log-transformed OR of type 2 diabetes and 1.13 (95% CI 1.04, 1.23; *p* = 0.004) for genetically predicted 10 mmHg increase in SBP.

Among lifestyle factors, higher genetically predicted coffee consumption was associated with an increased risk of senile cataract in the FinnGen consortium but not in the UK Biobank (Fig. 1). A suggestive association for genetically predicted coffee consumption remained in the meta-analysis (OR 1.18, 95% CI 1.00, 1.40; *p* = 0.050). Genetic predisposition to smoking initiation, but not genetically predicted alcohol consumption, was associated with senile cataract in both data sources (Fig. 1). The combined OR was 1.19 (95% CI 1.10, 1.29; *p* < 0.001) for genetically predicted one standard deviation increase in the prevalence of smoking initiation. The positive association between smoking initiation and senile cataract remained in the multivariable MR analysis with adjustment for alcohol consumption. Likewise, the null finding for alcohol consumption persisted after adjustment for smoking initiation.

Results of sensitivity analyses are presented in Table 2. The associations overall remained directionally consistent in sensitivity analyses albeit with wider CIs in the weighted median and MR-Egger methods. Most associations with the exception for the association for BMI in UK Biobank persisted in the contamination mixture model. We detected moderate heterogeneity in the analysis of type 2 diabetes and smoking initiation. However, we detected no pleiotropic effects in the MR-Egger regression (the *p* value of intercept > 0.1). Two, one and one outliers were observed in the analysis of BMI in FinnGen, smoking initiation in FinnGen and alcohol consumption in UK Biobank. These associations changed slightly after the removal of outliers.

**Table 2:** Associations of genetically predicted metabolic and lifestyle factors with senile cataract risk in sensitivity analyses.

Source	Exposure	SNPs	Cochrane’ Q	*p*_intercept_	Weighted median			MR-Egger			Contamination mixture		
					OR	95% CI	*p*	OR	95% CI	*p*	OR	95% CI	*p*
FinnGen	Body mass index	305	362	0.102	1.11	0.94, 1.31	0.200	0.99	0.77, 1.27	0.928	1.13	1.01, 1.28	0.049
FinnGen	Type 2 diabetes	472	552	0.652	1.08	1.01, 1.15	0.015	1.09	1.01, 1.17	0.025	1.06	1.02, 1.09	0.010
FinnGen	Systolic blood pressure	217	217	0.105	1.08	0.93, 1.24	0.314	0.82	0.56, 1.18	0.278	1.14	0.99, 1.3	0.078
FinnGen	Coffee consumption	12	12	0.444	1.33	1.01, 1.75	0.040	1.57	1.05, 2.34	0.051	1.31	1.02, 1.68	0.045
FinnGen	Alcohol consumption	80	104	0.279	0.86	0.55, 1.35	0.507	0.60	0.27, 1.33	0.213	0.64	0.4, 1.11	0.130
FinnGen	Smoking initiation	297	418	0.460	1.18	1.03, 1.36	0.018	1.00	0.65, 1.55	0.987	1.20	1.08, 1.34	0.003
UKBB	Body mass index	312	318	0.444	1.14	0.91, 1.44	0.243	1.04	0.75, 1.44	0.806	1.13	0.94, 1.32	0.216
UKBB	Type 2 diabetes	492	643	0.835	0.99	0.91, 1.08	0.814	1.01	0.91, 1.13	0.788	1.00	0.96, 1.05	0.999
UKBB	Systolic blood pressure	224	266	0.954	1.24	1.02, 1.53	0.035	1.19	0.68, 2.11	0.540	1.43	1.13, 1.92	0.004
UKBB	Coffee consumption	12	14	0.439	0.85	0.57, 1.28	0.441	1.06	0.59, 1.91	0.842	0.65	0.47, 1.13	0.191
UKBB	Alcohol consumption	84	117	0.219	1.05	0.55, 2.00	0.892	0.76	0.34, 1.71	0.513	1.52	0.9, 4.44	0.130
UKBB	Smoking initiation	312	385	0.544	1.13	0.95, 1.35	0.162	1.42	0.82, 2.46	0.211	1.27	1.03, 1.49	0.028

*CI* confidence interval, *OR* odds ratio, *SNPs* single nucleotide polymorphisms, *UKBB* UK Biobank.

## Discussion

The present MR study found that higher genetically predicted BMI and SBP and genetic predisposition to type 2 diabetes and smoking initiation were associated with an elevated risk of senile cataract in a combined sample of 26,489 cases and 509,767 non-cases. There was a suggestive increased risk of senile cataract with higher genetically predicted coffee consumption, but no evidence of an association with genetically predicted moderate alcohol consumption.

Our MR findings concerning the associations of metabolic syndrome components with senile cataract supported previous observational findings. In a Swedish cohort of 35,369 women followed up for a mean of 8.2 years, the metabolic syndrome and its components, abdominal adiposity, diabetes, and hypertension were associated with an increased risk for cataract extraction^5^. These associations were subsequently replicated in another cohort study of 45,049 Swedish men^6^. Meta-analyses based on observational studies also found that an elevated BMI^33^, type 2 diabetes^34^ and hypertension^35^ appeared to increase the risk of senile cataract. The present study based on genetic data confirmed and strengthened the causality of these associations, which indicated an increased risk of cataract among individuals with these metabolic risk factors. Therefore, maintaining a healthy BMI and blood pressure can now be more strongly recommended as prevention strategies for senile cataract. With regard to diabetes, although we observed a positive association with cataract, the magnitude of this association should be interpreted with caution given that the MR estimate was based on genetic liability to diabetes. Thus, we might have underestimated this association compared to the observational findings^34^. However, this finding still confirmed the causal detrimental role of diabetes in the development of senile cataract, which suggests that reducing the prevalence of diabetes from the population perspective and doing good diabetes management (e.g., maintaining a healthy glycemic profile) from the clinical perspective may benefit the senile cataract prevention.

Epidemiological data on the association between cigarette smoking and senile cataract are consistent and reveal an increased risk of senile cataract in both current and past smokers compared to never smokers^13,36,37^. This MR study confirmed the causal impact of smoking on senile cataract. Studies also showed smoking cessation as an efficient practice to lower the high risk of senile cataract in smokers even though it may take a long time for the increased risk to decline in heavy smokers^36–38^. Taken together, reducing the prevalence of smoking initiation appears to be an applicable way to decrease smoking-raised burden of senile cataract once and for all, and for current smokers smoking cessation should be promoted.

Studies on coffee consumption in relation to senile cataract are scarce and conflicting. A zone-level ecological study revealed an inverse correlation between coffee consumption and prevalence of senile cataract^9^. However, no association between coffee consumption and senile cataract was observed in a cross-sectional study^14^. Results of our study showed a positive association between genetically predicted coffee consumption and senile cataract in the Finnish population, whereas this association was not replicated in the UK Biobank study. Given these inconsistent data, more research is warranted to examine the association between coffee consumption and senile cataract.

Alcohol consumption has been inconsistently associated with senile cataract in observational studies. In a cohort of 34,713 Swedish women, the risk of senile cataract started to increase at a daily use of ≥ 1 alcoholic drinks^10^. However, a recent study with data from two large cohorts implied that low-to-moderate alcohol consumption might lower the risk of undergoing cataract surgery^39^, which is in line with a meta-analysis including observational studies published before May 2014^11^. In another meta-analysis based on 7 prospective cohort studies, the pattern of association between alcohol consumption and senile cataract risk was unclear^12^. This MR study did not detect an association between genetically predicted moderate alcohol consumption and senile cataract, which might hint that the discrepancy across above studies might be attributed to residual confounding.

There are several strengths of the present study. The major merit was the MR design, which minimized residual confounding and other biases and thus reinforced the causal reference in the associations of metabolic and lifestyle traits with risk of senile cataract. We confined the study population to individuals of European ancestry in all analyses with the exception for that of type 2 diabetes. Associations were examined in two independent data sources.

There are several limitations of the present study. MR studies can be biased by horizontal pleiotropy. However, we found limited evidence indicating the presence of horizontal pleiotropy based on consideration of the intercept in the MR-Egger regression, few outliers identified by the MR-PRESSO method and consistent causal estimates from sensitivity analyses. SNPs for type 2 diabetes were extracted from a genome-wide association study where less than 21% of the populations are non-European participants^18^, which might have introduced population structure bias in the analysis. However, the proportion of non-European individuals was small and genetic principal components were adjusted for in the genome-wide association studies^18^. Thus, our findings are not likely affected by this bias. There was sample overlap in certain analyses in the UK Biobank. The sample overlap might make the model overfitting and bias the causal estimate towards the observational estimate^40^. Nevertheless, this sample overlap was less likely to mislead our results since our genetic instruments were selected from large-scale genome-wide association studies and we interpreted associations based on the results of meta-analysis of FinnGen and UK Biobank where most of the weight came from FinnGen, which included 3 times as many cases as UK Biobank. In addition, the F statistics of all associations with the exception for that of alcohol consumption in FinnGen (no sample overlap) were over 10, which indicated a good strength of used genetic instruments and no weak instrument bias^40^. Given that senile cataract usually onsets at an old age and shares risk factors with other diseases, like cancer and cardiovascular disease, our MR estimates might be risked by survivor bias^41,42^. However, two populations included in this study were generally young and possibly less likely to be influenced. We could not completely rule out the possibility that selected SNPs are not associated with any confounders (i.e., the violation of the independence assumption of MR) and therefore the observed associations might be challenged by confounding. The confinement of study population to individuals of European descent might limit the generalizability of our findings to other populations. This two-sample MR study was based on several genome-wide association studies in different populations. Differences in population structures, such as female to male ratio and age distribution, might bias our findings. In addition, information on subtypes of cataract was unavailable, and thus, whether the effects of metabolic and lifestyle factors differ across cataract types needs investigation^7^. For analyses of alcohol and coffee consumption, non-linear association was unlikely to be examined in the present study based on the summary-level data.

In conclusion, this study suggests that obesity, type 2 diabetes, high SBP and smoking may be causally associated with the risk of developing senile cataract. Whether coffee consumption increases the risk of senile cataract needs verification. Moderate alcohol consumption appears not to be a causal risk factor for senile cataract.

## Data Availability

Data from UK Biobank (http://www.nealelab.is/uk-biobank) and FinnGen consortium (https://r4.finngen.fi/pheno/H7_CATARACTSENILE) are publicly available. All data analyzed in this study are available in OSF data respiratory (https://osf.io/by3un/).
